# Multiple Intimal Injuries Associated With Severe Coronary Spasm

**DOI:** 10.1002/ccr3.72050

**Published:** 2026-02-15

**Authors:** Koichiro Hori, Riku Arai, Keisuke Kojima, Yasuo Okumura

**Affiliations:** ^1^ Division of Cardiology, Department of Medicine Nihon University School of Medicine Tokyo Japan

**Keywords:** intimal injury, optical coherence tomography, sudden death, vasospasm

## Abstract

Coronary vasospasm is a recognized but underappreciated cause of out‐of‐hospital cardiac arrest. However, the intracoronary imaging features associated with life‐threatening vasospastic events have not been fully characterized. We present a patient who experienced recurrent cardiac arrest due to severe coronary vasospasm. Optical coherence tomography (OCT) revealed multiple focal intimal injuries, microthrombus formation, and vessel shrinkage, despite the absence of fixed coronary obstruction. The patient developed repeated ischemic events and ultimately died from nonocclusive mesenteric ischemia despite intensive care. This case illustrates that severe coronary vasospasm can cause repeated intimal injury and trigger fatal cardiac arrest. OCT may help identify a high‐risk vasospastic phenotype that requires heightened clinical vigilance and aggressive vasodilator‐based management.

## Key Clinical Message

Multiple coronary intimal injuries detected by optical coherence tomography (OCT) may represent a severe vasospastic phenotype associated with recurrent ischemic events and sudden cardiac arrest. Recognition of this OCT pattern may help identify high‐risk patients who require heightened clinical vigilance and aggressive vasodilator therapy to prevent fatal complications.

## 1 Case Image Presentation

**FIGURE 1:**
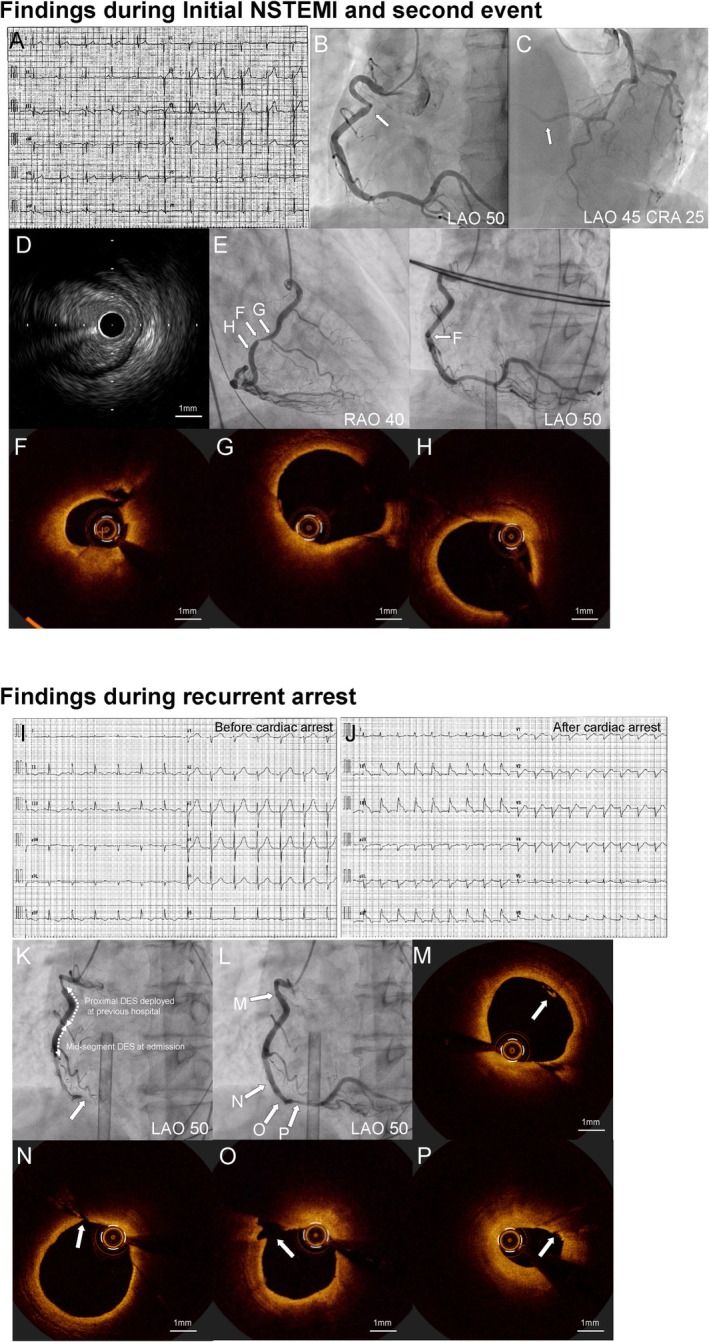
(A) Initial electrocardiogram at first presentation. (B) Coronary angiography demonstrating right coronary artery (RCA) narrowing. (C) Collateral flow from the left coronary artery to the RCA, suggesting transient occlusion. (D) Intravascular ultrasound showing plaque‐rich stenosis in the RCA. (E) Coronary angiography revealing severe mid‐RCA narrowing during the second event. (F) Optical coherence tomography (OCT) demonstrating localized fibrous cap disruption in the mid RCA. (G, H) Adjacent RCA segments with preserved vessel architecture on OCT. (I, J) Electrocardiograms obtained around the time of recurrent cardiac arrest. (K) Coronary angiography showing distal RCA occlusion during the final event. (L) Restoration of distal RCA flow after intracoronary isosorbide dinitrate administration. (M) OCT showing microthrombus formation (arrow). (N–P) OCT demonstrating multiple focal intimal injuries and vessel shrinkage (arrows).

A 60‐year‐old man with no history of smoking, illicit drug use, or hypertension presented with non–ST‐segment elevation acute coronary syndrome (ACS) (Figure 1A) and underwent percutaneous coronary intervention, after which standard postpercutaneous coronary intervention (PCI) medical therapy including dual antiplatelet therapy and a statin was initiated. Coronary angiography (CAG) demonstrated collateral flow from the left coronary artery (LCA) to the right coronary artery (RCA), suggesting transient occlusion or severe spasm, although no fixed obstruction was seen (Figure 1B,C). Intravascular ultrasound (IVUS) revealed plaque‐rich stenosis (Figure 1D), treated with drug‐eluting stent (DES) placement.

One month later, he experienced ventricular fibrillation and required venoarterial extracorporeal membrane oxygenation (VA‐ECMO). CAG revealed severe mid‐RCA narrowing, which did not respond to 1 mg of isosorbide dinitrate (ISDN) (Figure 1E). Optical coherence tomography (OCT) demonstrated fibrous cap disruption in the mid RCA, with otherwise normal adjacent segments. (Figure 1F–H). Although OCT did not show clear plaque rupture, localized fibrous cap disruption was present and considered contributory to recurrent ACS, and PCI with DES implantation was performed. Continuous intravenous ISDN was administered post‐PCI. Following neurological recovery under targeted temperature management (TTM), VA‐ECMO was successfully weaned on day 3, with no significant ECG changes during TTM.

Three hours after ECMO removal, he developed ST‐segment elevation (Figure 1I,J), followed by pulseless electrical activity, necessitating emergent VA‐ECMO reinstitution. CAG showed distal RCA occlusion relieved after 15 mg ISDN (Figure 1K,L). OCT revealed microthrombus, multiple intimal injuries, and vessel shrinkage (Figure 1M–P). Following this event, intensive oral vasodilator therapy, including benidipine and nicorandil, was initiated. The patient had already been under general anesthesia since the second event, using remifentanil, midazolam, and rocuronium. Despite aggressive care, he died on day 4 from nonocclusive mesenteric ischemia, likely secondary to vasospasm.

## 2 Clinical Question

In a patient with recurrent cardiac arrest and transient coronary occlusion, what mechanism explains the repeated events and the multiple coronary intimal injuries observed on OCT?

## 3 Discussion

This patient experienced three ACS episodes in a short period, two at rest in the early morning. In the first event, the collateral flow suggests transient occlusion, possibly due to resolved thrombus or severe spasm. Intimal rupture has been documented in vasospastic angina; however, the extensive and multiple lesions identified in our case on final‐event OCT may represent a more severe phenotype that has not been well characterized in previous literature [1, 2, 3].

Although a “pearl‐string” appearance on angiography reflects dynamic coronary spasm, the corresponding OCT morphology is variable. Multiple extensive intimal injuries, as observed in our case, have not been consistently reported and may represent an unusually severe manifestation of vasospastic injury. The focal distribution of intimal injuries on OCT, along with the rapid angiographic response to intracoronary ISDN, makes global hypoxic injury unlikely as a confounding factor. Systemic causes of coronary spasm, including connective tissue and rheumatologic diseases such as scleroderma, were evaluated by comprehensive serological screening and were considered unlikely.

Importantly, this case highlights the diagnostic advantage of OCT over conventional CAG. While angiography primarily depicts luminal changes, OCT provides high‐resolution visualization of the vessel wall, enabling detection of subtle intimal injuries, fibrous cap disruption, and microthrombus formation that may not be apparent on angiography alone. In the setting of severe coronary vasospasm, OCT can therefore offer critical mechanistic insights and help identify a high‐risk phenotype that might otherwise be underestimated.

These findings underscore that severe coronary vasospasm can manifest as multiple intimal injuries on OCT, carry a high risk of sudden cardiac arrest, and require close clinical attention. OCT plays a crucial diagnostic role in identifying this high‐risk phenotype and guiding appropriate vasodilator‐based management.

## Author Contributions

**Koichiro Hori:** conceptualization, investigation, writing – original draft, writing – review and editing. **Riku Arai:** writing – original draft, writing – review and editing. **Keisuke Kojima:** writing – review and editing. **Yasuo Okumura:** supervision, writing – review and editing.

## Funding

The authors have nothing to report.

## Disclosure

Dr. Koichiro Hori received an honorarium from Asahi Intecc Co. Ltd.

## Consent

Written informed consent was obtained from the patient for publication of this case report.

## Conflicts of Interest

The authors declare no conflicts of interest.

## Data Availability

The data underlying this article are available in the article.
